# Impairment of Motor Function Correlates with Neurometabolite and Brain Iron Alterations in Parkinson’s Disease

**DOI:** 10.3390/cells8020096

**Published:** 2019-01-29

**Authors:** Beate Pesch, Swaantje Casjens, Dirk Woitalla, Shalmali Dharmadhikari, David A. Edmondson, Maria Angela Samis Zella, Martin Lehnert, Anne Lotz, Lennard Herrmann, Siegfried Muhlack, Peter Kraus, Chien-Lin Yeh, Benjamin Glaubitz, Tobias Schmidt-Wilcke, Ralf Gold, Christoph van Thriel, Thomas Brüning, Lars Tönges, Ulrike Dydak

**Affiliations:** 1 Institute for Prevention and Occupational Medicine of the German Social Accident Insurance, Institute of the Ruhr University Bochum (IPA), 44789 Bochum, Germany; pesch@ipa-dguv.de (B.P.); casjens@ipa-dguv.de (S.C.); lehnert@ipa-dguv.de (M.L.); lotz@ipa-dguv.de (A.L.); bruening@ipa-dguv.de (T.B.); 2 Department of Neurology, St. Josef-Hospital, Ruhr-University Bochum, 44791 Bochum, Germany; d.woitalla@contilia.de (D.W.); lennard.herrmann@rub.de (L.H.); siegfried.muhlack@rub.de (S.M.); peter.kraus@rub.de (P.K.); ralf.gold@rub.de (R.G.); lars.toenges@rub.de (L.T.); 3 Department of Neurology, St. Josef-Hospital, Katholische Kliniken Ruhrhalbinsel, Contilia Gruppe, 45257 Essen, Germany; maria.zella@rub.de; 4 School of Health Sciences, Purdue University, West Lafayette, IN 47907, USA; stdharm@emory.edu (S.D.); edmondsd@purdue.edu (D.A.E.); ln7511@gmail.com (C.-L.Y.); 5 Department of Radiology and Imaging Sciences, Indiana University School of Medicine, Indianapolis, IN 46202, USA; 6 Department of Radiology and Imaging Sciences, Emory University, Atlanta, GA 30322, USA; 7 Department of Neurology, BG University Hospital Bergmannsheil, Ruhr University Bochum, 44789 Bochum, Germany; benjamin.glaubitz@rub.de; 8 Department of Neurology, St. Mauritius Therapieklinik, 40670 Meerbusch, Germany; tobias-schmidt-wilcke@t-online.de; 9 Institute of Clinical Neuroscience and Medical Psychology, Universitätsklinikum Düsseldorf, 40225 Düsseldorf, Germany; 10 Leibniz Research Centre for Working Environment and Human Factors (IfADo), TU Dortmund, 44139 Dortmund, Germany; thriel@ifado.de

**Keywords:** Parkinson’s disease, brain iron, motor dysfunction, neurometabolites, magnetic resonance imaging, magnetic resonance spectroscopy, GABA, spectroscopy

## Abstract

We took advantage of magnetic resonance imaging (MRI) and spectroscopy (MRS) as non-invasive methods to quantify brain iron and neurometabolites, which were analyzed along with other predictors of motor dysfunction in Parkinson’s disease (PD). Tapping hits, tremor amplitude, and the scores derived from part III of the Movement Disorder Society-Sponsored Revision of the Unified Parkinson Disease Rating Scale (MDS-UPDRS3 scores) were determined in 35 male PD patients and 35 controls. The iron-sensitive MRI relaxation rate R2* was measured in the globus pallidus and substantia nigra. γ-aminobutyric acid (GABA)-edited and short echo-time MRS was used for the quantification of neurometabolites in the striatum and thalamus. Associations of R2*, neurometabolites, and other factors with motor function were estimated with Spearman correlations and mixed regression models to account for repeated measurements (hands, hemispheres). In PD patients, R2* and striatal GABA correlated with MDS-UPDRS3 scores if not adjusted for age. Patients with akinetic-rigid PD subtype (*N* = 19) presented with lower creatine and striatal glutamate and glutamine (Glx) but elevated thalamic GABA compared to controls or mixed PD subtype. In PD patients, Glx correlated with an impaired dexterity when adjusted for covariates. Elevated myo-inositol was associated with more tapping hits and lower MDS-UPDRS3 scores. Our neuroimaging study provides evidence that motor dysfunction in PD correlates with alterations in brain iron and neurometabolites.

## 1. Introduction

Parkinson’s disease (PD) is one of the most common movement disorders and is characterized by dopaminergic neurodegeneration primarily in the iron (Fe)-rich substantia nigra (SN) and α-synuclein pathology [1]. There is strong evidence that α-synuclein may act as cellular ferrireductase involved in the generation of bioavailable iron (Fe) [2]. Fe is the most abundant redox-active metal in the brain and subject to chelation in neuromelanin to avoid oxidative damage [3]. Brain Fe accumulation occurs in areas primarily associated with motor activity [4] and correlates with age and neurometabolite levels [5]. Studies in PD patients frequently found associations of nigral Fe with disease progression, including an impairment of gross motor functions [6,7,8,9]. A study in rhesus monkeys demonstrated that brain Fe accumulation was associated with an impaired fine motor function such as dexterity and motor speed [10]. A large prospective study showed that elevated systemic Fe can impair dexterity in healthy elderly men [11]. In PD patients, systemic Fe correlated with brain Fe [12], but less is known about the effects of brain Fe on fine motor functions. 

With the advent of neuroimaging, magnetic resonance spectroscopy (MRS) became available as non-invasive method to quantify neurometabolites, including neurotransmitters, as potential biomarkers of PD [13]. MRS allows for the estimation of low-molecular weight chemicals, such as *N*-acteylaspartate (NAA; a marker of neuronal function), total creatine (tCr; involved in energy metabolism), myo-inositol (mI; a glial cell marker), glutamate (Glu; an excitatory neurotransmitter), and glutamine (Gln; precursor of Glu and γ-aminobutyric acid (GABA)) [14]. Several MRS studies on PD exist, but due to differences in resolution and sensitivity, small sample sizes, and heterogeneous patient populations across the studies, results are rather heterogeneous [13,14].

Advanced MRS techniques, such as spectral editing, also enable the in vivo study of GABA, the primary inhibitory neurotransmitter in the central nervous system [15]. The pathophysiology of PD involves the indirect and direct pathways of motor control in the basal ganglia, which are neuronal circuits facilitating the initiation and execution of voluntary movement. These pathways depend on excitatory and inhibitory signaling and well-balanced regulation of the neurotransmitters dopamine, GABA, and Glu. Thus, exploring GABA and Glu neurotransmitters by MRS is of great interest to understand the disruption of motor pathways in PD, and their implications on gross and fine motor function. Recently, an association of brain neurotransmitters on gross motor function was demonstrated in PD patients [16]. Fine motor performance was predicted by thalamic GABA levels in a study on manganese-exposed workers, with manganese toxicity leading to a particular form of parkinsonism [17]. Less is known about the influence of neurometabolites, including GABA, on dexterity in PD.

The aim of this analysis was to explore associations between brain Fe, GABA, and neurometabolites involved in the energy metabolism, i.e., tCr, mI and the combined signal of Glu and Gln (Glx), with motor function in PD patients compared to controls within the framework of the study on WELDing and Oxidative damage (WELDOX II). We hypothesized that PD patients may also show associations of these neuroimaging variables with fine motor function. Furthermore, we evaluated differences in Parkinson clinical phenotypes.

## 2. Materials and Methods

### 2.1. Ethics Statement

This study protocol was approved by the ethics committee of Ruhr University Bochum (registration number 4762-13) and conducted according to the principles expressed in the Declaration of Helsinki. All participants provided written informed consent. 

### 2.2. Study Groups

For this analysis, we investigated 35 male PD patients and 35 male controls of similar age with complete neuroimaging data and variables from various motor performance tests. Subjects were recruited for the neuroimaging study WELDOX II from 2013 to 2016, as previously described [5,18,19]. Eligible for the present analysis were right-handed men, who did not suffer from alcohol abuse or claustrophobia. 

Education, smoking habits, and other characteristics were assessed by questionnaires in face-to-face interviews. The analysis of blood samples was performed with methods as formerly described [5,18,20]. Carbohydrate-deficient transferrin >2.6% was presumptive for alcohol abuse, which may affect Fe metabolism and motor functions.

### 2.3. Diagnosis of PD, the Hoehn and Yahr Scale, and MDS-UPDRS 3 Scores

PD patients were enrolled at the Department of Neurology, St. Josef-Hospital, Bochum, Germany, and were diagnosed according to the criteria of the UK Parkinson Disease Society Brain Bank [21]. Disease severity was assessed by a neurologist (DW, SM, LH) using the Movement Disorder Society-Sponsored Revision of the Unified Parkinson Disease Rating Scale (MDS-UPDRS, part I–III) [22] and the Hoehn and Yahr scale [23]. All assessments reported in this study were performed with patients being on their regular medication. Patients on GABAergic medication were ineligible for participation. 

The MDS-UPDRS part III scores (further referred to as MDS-UPDRS3 total scores) were additionally verified—except for the rigidity subscore—by an independent rater based on video documentation of motor functions in all participants with the permission of the subject and blinded for disease status (DW, MZ). An MDS-UPDRS3 motor rigidity score (further referred to as MDS-UPDRS3 rigidity subscore) was defined as the sum of rigidity measures of the neck, the right and left upper extremity and the right and left lower extremity. Each motor function was rated with 0–4 points, resulting in a range from 0 to 20 for the summary subscore. In the present analysis, we included only patients with an akinetic-rigid (*N* = 19) or mixed subtype (*N* = 16) [16].

### 2.4. Fine Motor Tests

All fine motor tests were performed while the patients were on medication. Dexterity was tested for the right and left hand with a tapping test. Tapping hits were determined with the Motor Performance Series (Schuhfried, Mödling, Austria) as previously described [11]. The number of hits, a measure of motor speed, was acquired by tapping a stylus within 32 s as often as possible on a 1600 mm^2^ plate. Average tremor amplitude was calculated from drawn spirals (participants were asked to track a given spiral) and gave a measure of kinetic tremor [24].

### 2.5. MRI and MRS Data Acquisition and Processing 

MRI scans were performed on a 3 T Philips Achieva X-series whole-body clinical scanner (Philips Healthcare, Best, The Netherlands) with a 32-channel head coil as previously described [5,18]. In brief, R2* (= 1/T2*) relaxation rates were measured using a high-resolution (isotropic 1.5 mm^3^) 3D fast field echo (FFE) sequence with multiple flip angles (repetition time (TR) = 24.3 ms, echo time (TE) = 3.7 ms, ΔTE = 4.4 ms). R2* regions of interest (ROIs) were manually placed bilaterally in the substantia nigra (SN) and the globus pallidus (GP). 

For the quantification of neurometabolites, 30 mm × 30 mm × 25 mm voxels of interest (VOIs) were centered on the thalamus and the striatum (head of caudate nucleus, putamen and part of GP interna) of both hemispheres. Both short echo time point resolved spectroscopy (PRESS) spectra (TE/TR = 30/2000 ms, 32 averages) and MEGA-PRESS edited GABA spectra (TE/TR = 68/2000 ms, edit ON acquisitions = 128, edit OFF acquisitions = 128) were acquired from each VOI [15]. In addition, reference spectra without water suppression were obtained for phase, frequency, and eddy current correction. Brain tissue segmentation into gray matter (GM), white matter (WM), and cerebrospinal fluid (CSF) was performed using the partial volume correction tool by Nia Goulden and Paul Mullins (https://www.bangor.ac.uk/psychology/biu/Wiki.php.en) with SPM8 as formerly described [25].

Post-processing and quantification of MRS data was done using LCModel (v 6.2-0R) [26] and was focused on GABA, Glx, tCr, and mI. Neurometabolite concentrations were referenced to the unsuppressed water signal. Tissue correction of neurometabolite content is applied to adjust for CSF in VOIs, assuming no metabolic activity in CSF. Only concentrations that were estimated with a relative standard deviation (%SD) <20%, as reported by LCModel, were used for further statistical analysis.

**Figure 1:**
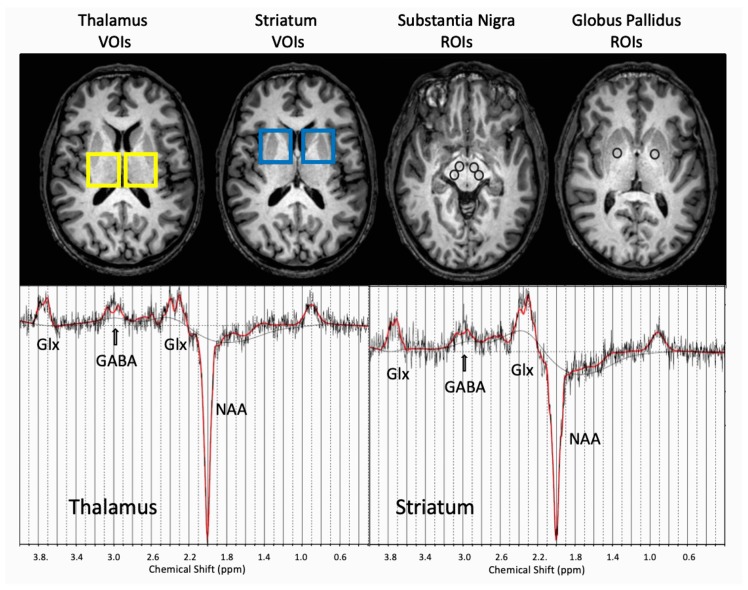
T1-weighted MRI images showing the placement of MRS volumes of interest (VOIs) centered on thalamus and striatum, and placement of regions of interest (ROIs) in the substantia nigra and globus pallidus for R2* analysis. Representative edited GABA spectra from the right thalamus and right striatum, indicating the LCModel fit (red solid line) with GABA, Glx, and NAA peaks shown below the MRI images. Abbreviations: T1: transverse relaxation time, MRI: magnetic resonance imaging, MRS: magnetic resonance spectroscopy, VOI: voxel of interest, ROI: region of interest, NAA: N-acetylaspartate, GABA: γ-aminobutyric acid, Glx: combined signal of glutamate and glutamine.

The placement of the MRI ROIs, MRS VOIs, and representative spectra are shown in Figure 1.

### 2.6. Statistics

For describing the distribution of neurometabolites, CSF-corrected concentrations (mM) were presented. For the analysis of associations, water-scaled values in institutional units (i.u.) were adjusted for the CSF content of the voxels by using partial correlation coefficients or by implementing CSF as covariate in the regression models. Neuroimaging data were presented with arithmetic means of measurements in both hemispheres for descriptive purposes, whereas the hemisphere was implemented as covariate in the regression models. 

Median and inter-quartile range (IQR) were used to describe the distribution of continuous variables. Group differences of continuous variables were tested with the Kruskal–Wallis or Wilcoxon rank-sum test and of categorical variables with the χ^2^ test. Spearman correlation coefficients (r_s_) were presented with 95% confidence intervals (CIs). 

We applied mixed models to fine motor tests (Poisson respectively loglinear regression) in all men and linear regression to MDS-UPDRS3 motor scores in PD patients for the association of neuroimaging data with motor dysfunctions, adjusted for CSF, and with PD subtype, age (in decades), education (high for university entrance level vs. lower levels), and the more affected hand as covariates. Education was a significant predictor of tapping and other fine motor tests that involve cognition or reaction time [11]. In addition, we also ran the models using CSF-corrected neurometabolites. Subjects were implemented as a random factor to account for repeated measurements (fine motor tests with both hands, neuroimaging in both hemispheres). Point estimates of these potential predictors were presented with β (additive in linear models) or exp(β) (factor in loglinear models) along with 95% CIs. Effects were considered significant when 0 (linear models) or 1 (loglinear models) was not included in the CIs. 

Two-sided *p*-values ≤ 0.05 are shown in bold, one-sided *p*-values ≤ 0.05 in italic, not corrected for multiple tests.

The calculations were performed with the statistical software SAS, version 9.4 (SAS Institute Inc., Cary, NC, USA).

## 3. Results

### 3.1. Demographics and Clinical Data of the Study Population

Characteristics of this right-handed male study population are shown by the study group in Table 1. Median age at PD diagnosis was 54 (IQR 49–60) years for all patients. Patients with akinetic-rigid PD were diagnosed at a slightly younger age than patients with a mixed PD clinical phenotype (52 vs. 55 years), had longer disease duration (6.0 vs. 3.5 years) and a lower level of education. 

PD patients presented with a median MDS-UPDRS3 total score of 34. Men with akinetic-rigid PD had similar total scores but higher rigidity subscores than those with mixed PD (median 7 vs. 4). Controls had MDS-UPDRS3 scores of 2 or lower (median 1). 

As compared with controls, PD patients showed larger tremor amplitudes (1.1 vs. 0.9 mm) and fewer tapping hits (164 vs. 186) with their left or more affected hand. Patients with akinetic-rigid PD performed slightly fewer tapping hits and smaller tremor amplitudes than those with mixed PD. 

Table S1 presents the motor variables for the right and left hand by potentially influencing factors, such as disease status, age, and education, which were implemented as predictors of motor function into the statistical models. In all strata, tapping with the right hand resulted in more hits than with the left hand (e.g., 212 vs. 186 in controls and 178 vs. 164 in all PD patients). This effect is less pronounced in PD patients where the right hand is the more affected side (176 vs. 171). A similar but weaker pattern for the results by hand can be observed for the amplitudes in spiral drawing. Subjects with a high school diploma performed more tapping hits, smaller tremor amplitudes and higher rigidity subscores than men with a lower education. 

Table S2 shows that there were no obvious correlations between gross and fine motor dysfunctions in PD patients (e.g., between MDS-UPDRS3 rigidity subscore and tapping r_s_ = 0.14, 95% CI −0.20–0.45) but depicts a weak negative association between tapping hits and tremor amplitude (r_s_ = −0.32, 95% CI −0.59–0.02) and a strong positive correlation between MDS-UPDRS3 total and rigidity subscores (r_s_ = 0.78, 95% CI 0.61–0.89).

### 3.2. Distribution of Brain Iron and Neurometabolites

Table 2 presents the distribution of R2* in SN and GP, as well as the distribution of the CSF-corrected neurometabolites in the striatum or the thalamus with median and IQR values. There were no obvious group differences of R2* values in these ROIs. PD patients showed lower striatal tCr than controls (7.2 vs. 7.5 mM), and akinetic PD patients had lower striatal tCr than mixed PD patients (6.7 vs. 7.4 mM). Akinetic PD patients also displayed lower striatal Glx levels compared to mixed PD (9.6 vs. 11.4 mM), with overall similar concentrations of 10.5 mM in all PD patients and controls. No group difference was found for local GABA concentrations. Box plots of the distributions of striatal and thalamic GABA, Glx, mI and tCr are displayed in Figure S1. 

### 3.3. Correlations Between Neuroimaging Data and Motor Dysfunctions

The correlations between neuroimaging data and motor dysfunctions in PD patients are depicted in Table 3. Nigral and pallidal R2* correlated with both the MDS-UPDRS3 total scores and the rigidity sub-scores (e.g., R2* in SN with total score: r_s_ = 0.39, 95% CI 0.07–0.64). Likewise, increased striatal GABA correlated with worse motor variables (e.g., with MDS-UPDRS3 total score: r_s_ = 0.37, 95% CI −0.08–0.69), although with wide confidence limits. Higher striatal mI was associated with more tapping hits (r_s_ = 0.49, 95% CI 0.06–0.77). Scatter plots for these associations are displayed in Figure S2.

### 3.4. Predictors of Impaired Fine Motor Functions

Predictors of fine motor tests were estimated in all subjects as shown in Table 4 displaying impaired dexterity, especially in PD patients with mixed subtype. After adjustment for CSF and other covariates, mI was positively associated with tapping hits (exp(β) = 1.27, 95% CI 1.06–1.53) in PD patients. Higher regional Glx levels, primarily in the thalamus, were associated with larger amplitudes in PD (exp(β) = 1.22, 95% CI 1.02–1.47). Similar associations were observed for the reported neurometabolites with direct CSF correction (data not shown). 

### 3.5. Predictors of Gross-Motor Symptoms

Finally, potential predictors of MDS-UPDRS3 total scores were estimated in PD patients, with age as strongest factor (β = 7.22, 95% CI 0.38–14.06) as demonstrated in Table 5. Low thalamic mI showed a notable association with higher scores (β = −29.00, 95% CI −58.21–0.21). The distribution of the rigidity subscores did not allow parametric regression modeling.

## 4. Discussion

Impairments in motor function are key clinical manifestations of PD and are hypothesized to be associated with changes in brain iron and neurometabolites, including those involved in energy metabolism and damage to dopaminergic neurons. The present analysis provides evidence of a positive correlation of nigral and pallidal R2* as well as striatal GABA with MDS-UPDRS3 total scores and rigidity subscores in all PD patients. After adjustment for covariates, lower mI levels predicted impaired gross and fine motor functions in PD, primarily in the thalamus. We further observed a positive association of thalamic Glx with larger tremor amplitudes. 

The present study found higher R2* values, representative of increased iron content, in the SN of PD patients and a correlation with gross motor function as evaluated with the MDS-UPDRS3, in line with results reported in recent studies [6,7] and several previous studies subjected to a narrative review on nigral Fe as biomarker of PD [9]. Using quantitative susceptibility mapping in addition to R2*-based iron mapping, the study by Langkammer et al. showed iron-based pathologic alterations also in GP and thalamus, as well as a correlation with PD severity. At higher resolution with 7T MRI, an association between R2*-weighted signals in clusters of dopaminergic cells within the SN and disease severity was found [27]. Importantly, our study shows that the correlation between R2* and MDS-UPDRS3 motor scales becomes weaker when adjusting for age because, in general, brain iron is known to increase with age [28,29]. Adjustment for age may therefore capture variance of an underlying causal association between iron accumulation and disease severity.

There is substantial variability of reported neurometabolite levels between MRS studies in Parkinson’s disease [13,14]. A major reason is the small size of these complex neuroimaging studies, which hinders the adjustment for pertinent covariates. Creatine plays a role in energy homeostasis by maintaining ATP levels constant in cells with high and fluctuating energy demands, primarily in muscles and glia [30]. In our study, patients with akinetic-rigid PD, an often clinically more severe phenotype of PD than the mixed or tremor-dominant phenotype, presented with lower striatal and thalamic tCr compared to the other phenotypes. Another analysis found basal ganglia tCr to be decreased in all PD phenotypes [31]. Therefore, using tCr as an internal reference for metabolite concentrations was inappropriate for this study, and internal water scaling was used instead, with adjusting for CSF fractions of the MRS VOI.

MDS-UPDRS3 scores strongly discriminated PD patients from controls. In contrast, differences in fine motor tests were less pronounced although PD patients displayed larger amplitudes and lower tapping speed than controls in line with other investigations [32,33]. In a large cohort of healthy subjects, we have demonstrated that age is a strong determinant of dexterity [11]. This was not as obvious in this study population, where the mixed PD phenotype and the left or more affected hand were stronger predictors. We observed lower tCr in PD patients as compared to controls, but could not observe an association with motor dysfunction. As already discussed for brain iron, implementing age into regression models may overadjust two parallel age-related processes, for example a putative correlation between tCr and motor dysfunction. Increasing age is correlated with both tCr levels [5,34] and motor dysfunction [11]. This may explain why we could not discern an association between tCr and fine motor function in our PD patients after adjustment for age.

Alterations in neurotransmission in PD are not restricted to the dopaminergic systems but importantly also involve the glutamatergic (glutamate and its precursor glutamine) and GABAergic networks [16]. Glx is an important precursor in the synthesis of GABA [35]. Furthermore, the cycling between Gln and Glu accounts for more than 80% of cerebral glucose consumption and is important for brain energetic metabolism [36]. In our study, akinetic-rigid PD patients exhibited significantly lower striatal Glx levels than the mixed PD phenotype, while there were no significant differences between the combined groups of PD patients and controls. However, better fine motor performance of PD patients was associated with lower Glx levels. O’Gorman et al. have shown that prefrontal Glx levels are associated with axial symptoms in PD, thus indicating that Glx seems to play a role in more complex motor function [16]. Furthermore, in our previous analysis, we had observed an age-related decline of Glx [5].

Non-MRS, invasive and ex-vivo techniques in animal models and postmortem human studies have established that the loss of dopaminergic striatal neurons in PD is accompanied by increased striatal GABA content [37,38,39,40]. In vivo GABA levels remain challenging to quantify due to the small MRS signal; however, several MRS studies at 3 T and higher magnetic fields have so far reported increased GABA levels in basal ganglia structures both in PD [16,41,42] as well as in manganese-exposed workers, who are at risk developing a form of parkinsonism [17,43]. Several of these studies also reported an association of basal ganglia GABA levels with gross motor function [16,42] or fine motor function as measured by a pegboard test [17]. The present study used statistical modeling, accounting for several confounding factors such as age. We found thalamic GABA levels to be slightly increased in the akinetic-rigid PD phenotype when compared to the mixed PD form. However, a correlation of striatal GABA levels with MDS-UPDRS3 scores showed wide confidence intervals and disappeared in the full prediction model. This is in line with a recent MRS study on manganese-exposed welders, that did not find any correlation of thalamic or striatal GABA levels with MDS-UPDRS3 scores, or with rigidity or tremor subscores, but showed that only age remained as predictor for the MDS-UPDRS3 scores in the statistical model [44]. 

Elevated levels of mI, a suggested marker for gliosis, in the brainstem and several cortical areas have been associated with neurodegenerative diseases [45], and have been shown to correlate negatively with gross motor function in ataxias and amyotrophic lateral sclerosis [46,47]. Notably, low systemic mI can increase blood glucose levels due to insulin-mimetic properties [48]. We found that lower mI levels in PD patients predicted motor dysfunctions, associated with less tapping hits and higher MDS-UPDRS3 total scores. This finding is in line with a previous report of decreased thalamic mI in manganese toxicity [49], which can lead to a Parkinsonian syndrome. In our previous analysis of mI in a mixed study population with healthy persons and PD or hematochromatosis patients, brain iron assessed with R2* in GP was a predictor of lower mI in these VOIs [5]. 

Overall, confidence limits of most estimates for associations between the study variables were rather wide, indicating additional influencing factors, such as potential effects of alcohol intake or body mass index on motor function in PD [50]. With recent reports on medication effecting both the on neurometabolites NAA and tCr [51] as well as on brain Fe levels and symptom scores [7], it is well possible that the medication status can modify the effects of neurometabolites or brain Fe on MDS-UPDRS3 scores. While patients receiving any GABAergic medication were ineligible for participation, nearly all patients were on dopaminergic treatment at the time of motor testing and neuroimaging. However, detailed information on medication, including a precise quantitative estimation of the levodopa equivalent dose and hours to time of investigation, were not available for all patients of this study. A further limitation of this and other costly neuroimaging studies is the small study size, which impairs a sound statistical modeling of the association between brain iron or neurometabolites with motor dysfunction allowing for the inclusion of pertinent confounders, effect modifiers, and interaction terms between study variables.

## 5. Conclusions

The present study provides evidence that brain iron and neurometabolite concentrations involved in energy metabolism and neurotransmission are associated with motor dysfunction in PD. To which extent neurometabolite alterations can be attributed to clinical PD phenotypes remains to be elucidated. 

## Figures and Tables

**Table 1:** Demographics and clinical data of male patients with Parkinson disease and of controls.

Participants Characteristics	Controls *N* = 35	PD *N* = 35	*p*-Value	Akinetic-Rigid PD *N* = 19	Mixed PD *N* = 16	*p*-Value
	Median (IQR)	Median (IQR)		Median (IQR)	Median (IQR)	
Age [years]	55 (50;66)	59 (54; 66)	0.14	60 (54; 67)	59 (55; 66)	0.64
Age at diagnosis [years]		54 (49; 60)		52 (47; 60)	55 (53; 59)	0.37
PD duration [years]		4.7 (2.5; 7.7)		**6.0 (3.6; 8.8)**	**3.5 (1.5; 5.4)**	**0.03**
MDS-UPDRS3 total score; range (0–132)	**1 (0; 2)**	**34 (24; 43)**	**<0.0001**	34 (24; 43)	34.5 (25.5; 45.5)	0.95
MDS-UPDRS3 rigidity subscore; range (0–20)	**0 (0; 0)**	**7 (3; 8)**	**<0.0001**	*7 (5; 8)*	*4 (2.5; 8)*	*0.10*
Tapping hits (left or more affected hand)	**186 (180; 205)**	**164 (134; 184)**	**0.0001**	163 (143; 178)	176 (126; 190)	0.80
Tremor amplitude (mm) (left or more affected hand)	**0.9 (0.7; 1.1)**	**1.1 (0.8; 1.3)**	**0.019**	1.1 (0.8; 1.2)	1.2 (0.8; 1.9)	0.26
	***N* (%)**	***N* (%)**		***N* (%)**	***N* %**	
Education						
Low	*8 (22.9)*	*17 (48.6)*	*0.08*	*12 (63.2)*	*5 (31.3)*	*0.06*
Medium	8 (22.9)	6 (17.1)		4 (21.1)	2 (12.5)	
High	19 (54.3)	12 (34.3)		3 (15.8)	9 (56.3)	
Clinically more affected side						
Left		12 (34.3)		5 (26.3)	7 (43.8)	0.24
Right		14 (40.0)		7 (36.8)	7 (43.8	
No preference		9 (25.7)		7 (36.8)	2 (12.5)	

Abbreviations: IQR: inter-quartile range (25th; 75th percentile), PD: Parkinson’s disease, MDS-UPDRS3: Movement Disorder Society-Sponsored Revision of the Unified Parkinson Disease Rating Scale part III, p-value obtained by Kruskal–Wallis test or χ^2^ test (significant effects marked in bold and marginal effects marked in italic).

**Table 2:** Distribution of brain iron and neurometabolites in male Parkinson patients and controls.

Neuroimaging Data	Controls *N* = 35	PD *N* = 35	*p*-Value	Akinetic-Rigid PD *N* = 19	Mixed PD *N* = 16	*p*-Value
	Median (IQR)	Median (IQR)		Median (IQR)	Median (IQR)	
R2* (1/s), SN (*N* = 70)	45.6 (40.8; 51.1)	48.0 (39.6; 61.8)	0.38	45.5 (40.4; 56)	53.4 (36.1; 62.4)	0.84
R2* (1/s), GP (*N* = 70)	43.4 (40.4; 47.7)	44.1 (37.9; 47.2)	0.43	39.9 (37.9; 46.2)	46.0 (38.2; 47.8)	0.41
Striatum						
GABA (mM) (*N* = 68)	1.9 (1.8; 2.1)	2.0 (1.8; 2.2)	0.30	2.0 (1.6; 2.2)	2.1 (1.9; 2.4)	0.13
Glx (mM) (*N* = 69)	10.5 (9.1; 11.3)	10.5 (8.9; 11.7)	0.88	**9.6 (8.8; 11.0)**	**11.4 (10.1; 12.3)**	**0.04**
Myo-inositol (mM) (*N* = 69)	4.3 (3.9; 4.7)	4.4 (3.9; 5.1)	0.37	4.3 (3.9; 4.8)	4.6 (4.0; 5.2)	0.30
Total creatine (mM) (*N* = 69)	**7.5 (7.1; 7.8)**	**7.2 (6.5; 7.6)**	**0.04**	**6.7 (6.4; 7.4)**	**7.4 (6.9; 7.8)**	**0.03**
Thalamus						
GABA (mM) (*N* = 69)	1.9 (1.7; 2.4)	2.1 (1.9; 2.4)	0.21	*2.2 (1.9; 2.4)*	*2.0 (1.9; 2.1)*	*0.06*
Glx (mM) (*N* = 69)	7.2 (6.6; 8.3)	7.7 (6.8; 8.5)	0.15	7.5 (6.8; 8.3)	7.8 (7.2; 8.9)	0.22
Myo-inositol (mM) (*N* = 69)	4.6 (4.2; 4.8)	4.9 (4.3; 5.4)	0.19	4.7 (3.9; 5.3)	5.0 (4.5; 5.5)	0.64
Total creatine (mM) (*N* = 69)	6.3 (6.1; 6.8)	6.3 (6.0; 6.8)	0.77	*6.2 (6.0; 6.5)*	*6.5 (6.2; 6.8)*	*0.06*

Abbreviations: PD: Parkinson’s disease, IQR: inter-quartile range (25th; 75th percentile) of the distribution using the arithmetic mean of both hemispheres, R: relaxation rate, SN: substantia nigra, GP: globus pallidus, GABA: γ-aminobutyric acid, Glx: glutamate and glutamine, p-value obtained by the Wilcoxon rank-sum test (significant effects marked in bold and marginal effects marked in italic).

**Table 3:** Spearman correlation coefficients with 95% confidence interval between age, neuroimaging data, and motor variables in 35 Parkinson patients.

r_s_ (95% CI)	Tapping Hits Non-dominant or More Affected Hand	Tremor Amplitude (mM) Non-Dominant Hand or More Affected Hand	MDS-UPDRS3 Total Score	MDS-UPDRS3 Rigidity Subscore
Age (years)	−0.10 (−0.42, 0.24)	0.09 (−0.25, 0.41)	**0.34 (0.01, 0.61)**	0.19 (−0.16, 0.49)
Age at diagnosis (years)	−0.11 (−0.43, 0.23)	0.17 (−0.18, 0.47)	0.26 (−0.08, 0.54)	0.07 (−0.27, 0.39)
R2* (1/s) SN	0.11 (−0.23, 0.43)	0.02 (−0.32, 0.35)	**0.39 (0.07, 0.64)**	*0.33 (−0.01, 0.59)*
R2* (1/s) GP	−0.04 (−0.37, 0.29)	0.00 (−0.34, 0.33)	*0.32 (−0.01, 0.59)*	*0.32 (−0.01, 0.59)*
Striatum				
GABA	0.31 (−0.14, 0.65)	0.13 (−0.32, 0.53)	*0.37 (−0.08, 0.69)*	0.30 (−0.15, 0.65)
Glx	−0.30 (−0.66, 0.16)	0.03 (−0.42, 0.47)	−0.14 (−0.55, 0.32)	−0.23 (−0.61, 0.24)
Myo-inositol	**0.49 (0.06, 0.77)**	*−0.34 (−0.68, 0.12)*	0.30 (−0.16., 0.66)	0.03 (−0.42, 0.47)
Total creatine	0.06 (−0.39, 0.49)	−0.29 (−0.65, 0.18)	0.14 (−0.32, 0.55)	0.01 (−0.44, 0.45)
Thalamus				
GABA	−0.11 (−0.55, 0.38)	−0.20 (−0.61, 0.30)	−0.01 (−0.47, 0.46)	−0.15 (−0.58, 0.34)
Glx	0.16 (−0.30, 0.56)	−0.03 (−0.47, 0.42)	0.15 (−0.31, 0.56)	0.11 (−0.35, 0.52)
Myo-inositol	−0.10 (−0.52, 0.36)	0.03 (−0.42, 0.47)	−0.19 (−0.58, 0.28)	−0.30 (−0.65, 0.17)
Total creatine	0.26 (−0.20, 0.63)	−0.01 (−0.45, 0.43)	0.01 (−0.44, 0.45)	−0.12 (−0.54, 0.34)

Abbreviations: MDS-UPDRS3: Movement Disorder Society-Sponsored Revision of the Unified Parkinson Disease Rating Scale part III, R: relaxation rate, SN: substantia nigra, GP: globus pallidus, GABA: γ-aminobutyric acid, Glx: glutamate and glutamine, rs: Spearman correlation coefficients (for neurometabolites partial correlation coefficients adjusted for CSF content in the voxels using data from the hemisphere contralateral to the non-dominant hand for tapping hits and tremor amplitude), CI: confidence interval (significant effects marked in bold and marginal effects marked in italic).

**Table 4:** Brain iron, neurometabolites, and other potential predictors of fine motor skills determined by a mixed model in 35 Parkinson patients and 35 controls.

	Tapping Hits			Tremor Amplitude (mm)		
	Exp(β)	95% CI		Exp(β)	95% CI	
Study groups (*N* = 70)						
Intercept	227.30	190.62	271.04	0.54	0.30	1.00
Akinetic-rigid PD vs. controls	**0.91**	**0.85**	**0.97**	1.06	0.87	1.30
Mixed PD vs. controls	**0.87**	**0.81**	**0.93**	**1.32**	**1.08**	**1.62**
High education vs. lower levels	**1.05**	**1.00**	**1.10**	0.95	0.80	1.12
Age [per 10 years]	*0.98*	*0.96*	*1.01*	1.06	0.96	1.18
Non-dominant hand	**0.90**	**0.86**	**0.95**	**1.18**	**1.12**	**1.25**
More affected side	**0.92**	**0.86**	**0.99**	**1.11**	**1.02**	**1.22**
						
Brain iron (*N* = 35 PD patients)						
R2* [log 1/s]	0.96	0.84	1.10	0.87	0.70	1.08
Globus pallidus vs. substantia nigra	0.99	0.94	1.06	0.98	0.92	1.04
						
Neurometabolites (*N* = 35 PD patients)						
GABA	1.01	0.85	1.22	1.03	0.84	1.25
Striatum vs. thalamus	0.99	0.91	1.07	0.97	0.89	1.05
						
Glutamate and glutamine	0.89	0.75	1.07	**1.22**	**1.02**	**1.47**
Striatum vs. thalamus	1.02	0.94	1.12	*0.93*	*0.85*	*1.01*
						
Myo-inositol	**1.27**	**1.06**	**1.53**	1.09	0.87	1.36
Striatum vs. thalamus	1.02	0.95	1.10	0.97	0.90	1.05
						
Total creatine	1.01	0.69	1.49	1.16	0.73	1.84
Striatum vs. thalamus	1.00	0.92	1.08	0.96	0.88	1.04

Abbreviations: PD: Parkinson’s disease, R: relaxation rate, GABA: γ-aminobutyric acid, exp(β): estimate of the regression coefficients (as factor of change per unit) of potential predictors of motor dysfunction in all subjects and for neuroimaging data in PD patients in separate models for each influencing factor adjusted for CSF content of the voxel (neurometabolites only), age (per 10 years), education (high, lower educational levels), hand (dominant, non-dominant hand), and affected side (more, less affected or no preference), CI: confidence interval (significant effects marked in bold and marginal effects marked in italic).

**Table 5:** Potential predictors of the MDS-UPDRS3 total scores in 35 Parkinson patients.

	Β	95% CI	
Intercept	−4.10	−46.71	38.51
Akinetic-rigid PD vs. mixed PD	−4.13	−14.40	6.14
High education	−4.71	−15.53	6.11
Age [per 10 years]	**7.22**	**0.38**	**14.06**
			
Brain iron			
R2*, globus pallidus [log 1/s]	19.72	−8.70	48.15
R2*, substantia nigra [log 1/s]	10.19	−7.41	27.78
			
Neurometabolites			
Striatum			
GABA	7.71	−21.54	36.95
Glutamate and glutamine	−5.48	−33.40	22.45
Myo-inositol	0.06	−31.11	31.23
Total creatine	14.59	−38.66	67.84
Thalamus			
GABA	−9.83	−46.20	26.54
Glutamate and glutamine	1.18	−28.92	31.28
Myo-inositol	*−29.00*	*−58.21*	*0.21*
Total creatine	−5.49	−75.39	64.42

Abbreviations: PD: Parkinson’s disease, GABA: γ-aminobutyric acid, R: relaxation rate, MDS-UPDRS3: Movement Disorder Society-Sponsored Revision of the Unified Parkinson Disease Rating Scale part III, β: estimate of regression coefficient (linear model) for potential predictors of motor dysfunction, in separate models for each neuroimaging variable adjusted for CSF content of the voxel (neurometabolites only), age (per 10 years), education (high, lower educational levels), CI: confidence interval (significant effects marked in bold and marginal effect marked in italic).

## Supplementary Materials

The following are available online at http://www.mdpi.com/2073-4409/8/2/96/s1: Figure S1: Box plots of the distributions of striatal and thalamic GABA, Glx, mI and tCr in 35 Parkinson patients and 35 controls; Figure S2: Correlations between brain iron (R2* placed in SN and GP) and GABA (in striatal and thalamic VOIs) with MDS-UPDRS3 total scores and of mI with tapping hits in 35 Parkinson patients; Table S1: Fine motor test results for both hands presented with medians for tapping hits and tremor amplitude in Parkinson patients and controls; Table S2: Spearman correlation coefficients with 95% confidence interval between motor functions in 35 Parkinson patients.
