# Supplementary material for: Impairment of Motor Function Correlates with Neurometabolite and Brain Iron Alterations in Parkinson’s Disease
Source: Cells. 2019 Jan 29;8(2):96. doi: 10.3390/cells8020096 (PMC6406520; doi:10.3390/cells8020096)
Supplement: Supplementary file 1 [file cells-08-00096-s001.pdf]

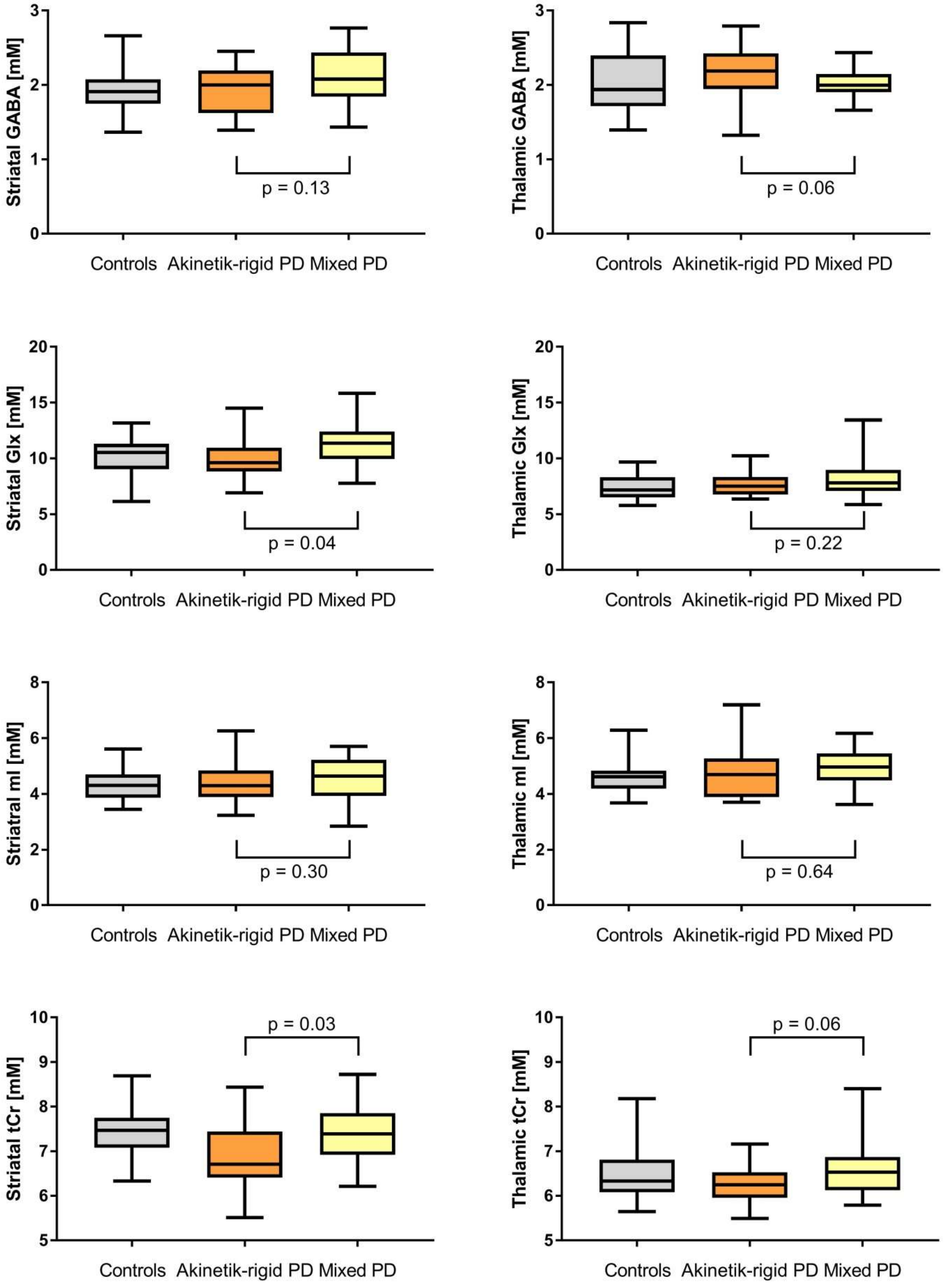

Figure 1. Box plots of the distributions of striatal and thalamic GABA, Glx, mI and tCr in 35 Parkinson patients and 35 controls. Abbreviations: GABA: γ-aminobutyric acid, Glx: glutamate and glutamine, mI: myo-inositol, tCr: total creatine, PD: Parkinson's disease, p value obtained by Kruskal-Wallis test.

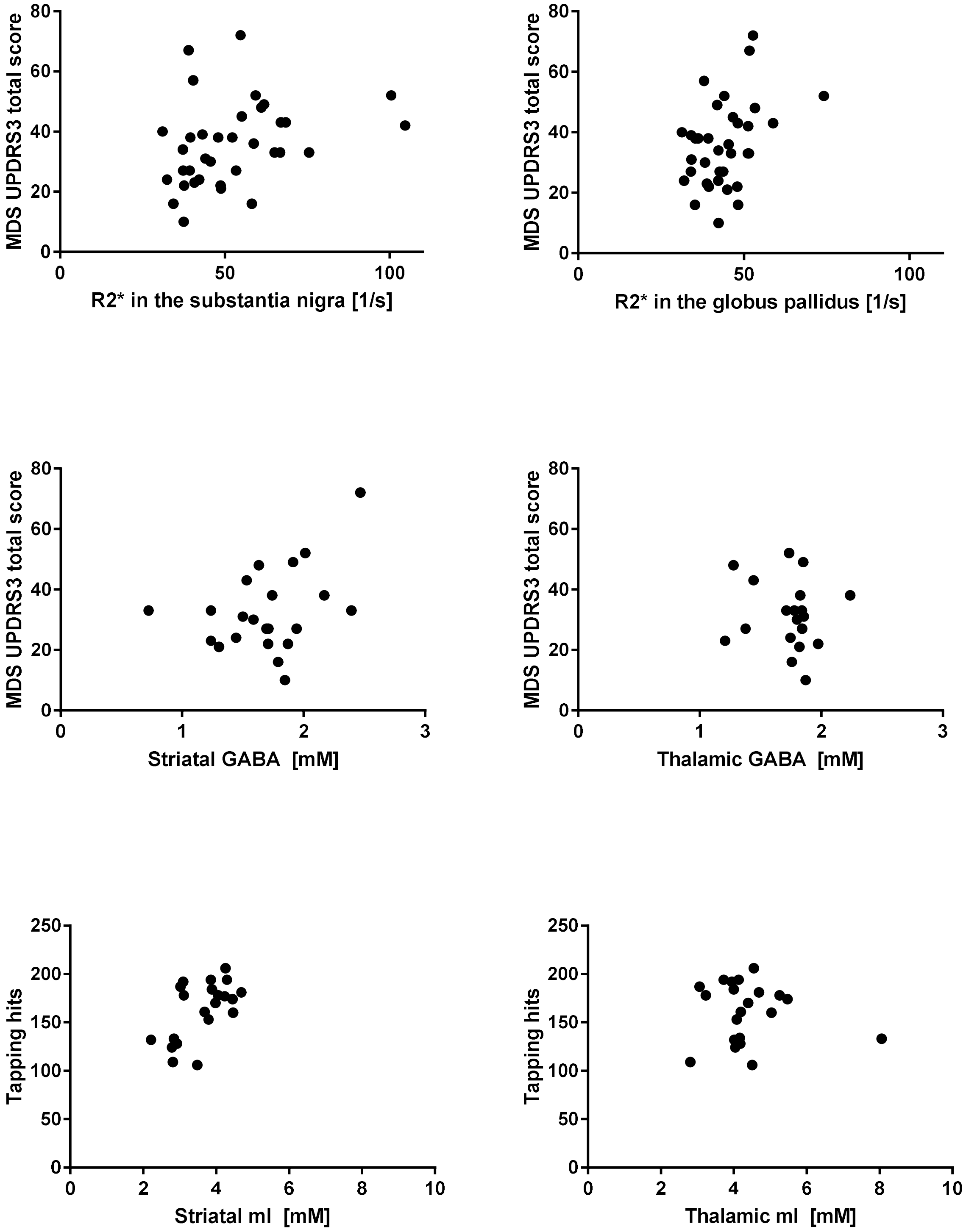

Figure S2. Correlatiations between brain iron (R2* in ROIs placed in SN and GP) and GABA (in striatal and thalamic VOIs) with MDS-UPDRS3 total scores and of mI with tapping hits in 35 Parkinson patients. Abbreviations: MDS-UPDRS3: Movement Disorder Society-Sponsored Revision of the Unified Parkinson Disease Rating Scale part III, R: relaxation rate, GABA: γ-aminobutyric acid, Glx: glutamate and glutamine, mI: myo-inositol, tCr: total creatine.

Table S1. Fine motor test results for both hands presented with medians for tapping hits and tremor amplitude in Parkinson patients and controls.

			**Tapping hits**		**Tremor amplitude [mm]**		**MDS-UPDRS3 total score**	**MDS-UPDRS3 rigidity subscore**
		**N**	**Left**	**Right**	**Left**	**Right**	**PD**	**PD**
**Total**		70	181	198	0.97	0.81	7.5	1.5
**Group**	Parkinson patients	35	164	178	1.17	0.92	34	7
	Controls	35	186	212	0.85	0.77	1	0
**Age**	≤ 58 years (median)	35	185	200	0.98	0.82	27	6
	> 58 years	35	179	187	0.95	0.81	38.5	7
**Education**	Low	25	166	186	1.10	0.87	39	7
	Medium	14	181	195	0.99	0.83	30.5	6
	High	31	194	203	0.82	0.81	35.5	4.5
**PD subtype**	Mixed	16	169	178	1.21	1.04	34.5	4
	Akinetic-rigid	19	164	182	1.12	0.89	34	7
**Clinically more**	Left	12	166	184	1.49	0.91	29	5.5
**affected side**	Right	14	171	176	0.81	0.83	31.5	5
**(PD)**	No preference	9	153	182	1.23	1.06	43	7

Abbreviations: PD, Parkinson's disease, MDS-UPDRS3: Movement Disorder Society-Sponsored Revision of the Unified Parkinson Disease Rating Scale part III.

Table S2. Spearman correlation coefficients with 95% confidence interval between motor functions in 35 Parkinson patients.

	**Tapping hits**			**Tremor amplitude [mm]**			**MDS-UPDRS3 total score**		
	r_S	95% CI		r_S	95% CI		r_S	95% CI	
Tremor amplitude [mm]	*-0.32*	*-0.59*	*0.02*						
MDS-UPDRS3 total score	0.08	-0.26	0.40	0.02	-0.31	0.35			
MDS-UPDRS3 rigidity subscore	0.14	-0.20	0.45	-0.13	-0.44	0.21	**0.78**	**0.61**	**0.89**

Abbreviations: PD: Parkinson's disease, MDS-UPDRS3: Movement Disorder Society-Sponsored Revision of the Unified Parkinson Disease Rating Scale part III, r_S: Spearman correlation coefficient, CI: confidence interval.
